# Outcomes in relation to antithrombotic therapy among patients with atrial fibrillation after percutaneous coronary intervention

**DOI:** 10.1371/journal.pone.0240161

**Published:** 2020-10-15

**Authors:** Jiesuck Park, Eue-Keun Choi, Kyung-Do Han, Bongseong Kim, You-Jung Choi, So-Ryoung Lee, Jeehoon Kang, Myung-Jin Cha, Kyung Woo Park, Seil Oh, Gregory Y. H. Lip

**Affiliations:** 1 Department of Internal Medicine, Seoul National University Hospital, Seoul, Republic of Korea; 2 Department of Biostatistics, College of Medicine, The Catholic University of Korea, Seoul, Republic of Korea; 3 Department of Statistics and Actuarial Science, Soongsil University, Seoul, Republic of Korea; 4 Liverpool Centre for Cardiovascular Science, University of Liverpool and Liverpool Chest & Heart Hospital, Liverpool, United Kingdom; 5 Department of Clinical Medicine, Aalborg Thrombosis Research Unit, Aalborg University, Aalborg, Denmark; China Medical University Hospital, TAIWAN

## Abstract

**Backgrounds:**

We investigated the prognostic impact of antithrombotic regimens at 1-year after percutaneous coronary intervention (PCI) among patients with atrial fibrillation (AF).

**Method and results:**

A total of 13,278 AF patients who underwent PCI from 2009 to 2013 were selected from Korean National Health Insurance Service database. Patients were categorized by antithrombotic regimens at 1-year after PCI: (1) OAC with or without single antiplatelet (OAC±SAPT); (2) triple therapy (TT) and (3) antiplatelets (APT) only. After propensity score matching, composite ischaemia (death, myocardial infarction, and stroke), composite bleeding (intracranial hemorrhage and gastrointestinal bleeding), and a composite clinical outcome (composite ischaemia and bleeding) were compared. Of total population, 1,100 (8.3%), 746 (5.6%), and 11,432 (86.1%) were treated with OAC±SAPT, TT, and APT only, respectively. Compared to OAC±SAPT group, the TT group had significantly higher risk of the composite clinical outcome (hazard ratio [HR] 1.46, 95% confidence interval [CI] 1.00–2.13) attributed to a higher trend in both ischaemia (HR 1.63, 95% CI 0.99–2.67) and bleeding (HR 1.22, 95% CI 0.69–2.13). The APT only group showed a higher risk of ischaemia (HR 1.85, 95% CI 1.25–2.74), despite a lower risk of bleeding (HR 0.55, 95% CI 0.32–0.94) compared to OAC±SAPT group.

**Conclusions:**

OAC±SAPT was associated with better clinical outcomes compared to TT or APT only treatments, beyond 1-year after PCI among Asians with AF.

## Introduction

Patients with atrial fibrillation (AF) at moderate to high risk of stroke are recommended for stroke prevention with oral anticoagulants (OAC) and for those who underwent percutaneous coronary intervention (PCI), combination antithrombotic therapy with OAC and antiplatelets (APT) is required [1–3]. However, previous studies reported an under-prescription of OAC in patients with AF after PCI, especially among the Asian population [4–7]. This relates to concerns that Asians with AF tend to have a higher risk of stroke and more seriously, intracranial hemorrhage when compared to non-Asians [8]. Also, those on OAC were associated with a higher rate of major bleeding than non-Asians [9]. It is therefore important to determine the optimal regimen and duration of antithrombotic therapy among the Asian patients with AF after PCI. Recently, the Optimizing Antithrombotic Care in Patients With AtriaL fibrillatiON and Coronary stEnt (OAC-ALONE) trial was conducted comparing the efficacy and safety benefit of OAC alone versus dual therapy (OAC plus SAPT) among Asian patients with AF beyond 1-year after PCI [10]. However, the study was underpowered for the primary outcome due to early trial termination related to delayed enrollment of study population. In real-world clinical practice, the optimal antithrombotic therapy regime in the period after PCI among patients with AF is important given the underuse of OAC [9] and early discontinuation of APT [11]. Therefore, we sought to investigate the treatment patterns and the prognostic impact of different antithrombotic therapy regimes on ischaemic and bleeding events, at 1-year after PCI among patients with AF.

## Methods

Study population and clinical data were derived from the National Health Insurance Service (NHIS) claim database of Korean population which includes inpatient and outpatient medical records, diagnostic codes and claims for the procedure or medication prescription [12, 13]. For the current study, a total of 226,118 patients who had PCI with coronary stent implantation between 2009 to 2013 were screened for inclusion (S1 Fig). Procedure codes for PCI and coronary stent implantation (M6561, M6562, M6563, and M6564) were used as PCI events. Of them, we excluded patients with death, myocardial infarction (MI) or PCI procedure within 1 year after the index procedure, those without AF diagnosis, those with incomplete prescription records or no antithrombotic therapy at 1-year after PCI. Patients with AF were defined as those with diagnostic codes for AF (International Classification of Disease, Tenth Revision, Clinical Modification [ICD-10-CM] I480–I484 and I489) at ≥1 hospitalization or at ≥2 outpatient clinics. To exclude patients with AF associated with valvular heart disease, those with diagnostic codes for mitral stenosis (I50, I52, and I59) or mechanical heart valves (Z952–Z954) were excluded. Finally, a total of 13,278 patients with non-valvular AF and complete prescription records were included for analysis. The definitions of comorbidities were summarized in S1 Table and have been validated in previous studies [5, 14, 15]. In brief, hypertension and diabetes mellitus (DM) were defined as diagnostic codes with claims of at least one prescription of antihypertensive or antidiabetic drug respectively. Patients with previous history of congestive heart failure (CHF), stroke or systemic thromboembolism, myocardial infarction (MI), peripheral artery disease (PAD), and intracranial hemorrhage (ICH) were defined based on ICD-10-CM codes. Individual stroke risk was estimated by the CHA_2_DS_2_-VASc score. This study was approved by the Institutional Review Board of Seoul National University Hospital (1706-160-863). The informed consent was waived by the review board because the identification number of each patient in the NHIS database is de-identified and encrypted to protect patient privacy.

Inpatient and outpatient records for antithrombotic therapy including aspirin, clopidogrel, vitamin K antagonists (VKA), and non-vitamin K antagonist oral anticoagulants (NOAC) were retrieved within 3-months windows before and after the 1-year follow-up period from the index PCI event. New generation P2Y_12_ inhibitors including prasugrel and ticagrelor were not considered as APT because they were not widely available in Korea during the study period. To reduce misclassification in the treatment group, patients with claims for at least 3-month prescription of antithrombotic therapy (OAC or APT) within the window periods were considered as those who were on treatment. For further comparison of clinical outcomes, patients were categorized in to three groups based on prescription regimen of antithrombotic therapy: OAC monotherapy and OAC + SAPT (OAC ± SAPT), triple therapy (TT, OAC + DAPT), and APT only (SAPT or DAPT).

For ischaemic risks, the composite ischaemic outcome was defined including MI, ischaemic stroke or death. For bleeding risk, the occurrence of ICH or gastrointestinal bleeding (GIB) were combined into composite bleeding outcome. Also, a composite clinical outcome was defined comprising composite ischaemic and bleeding outcomes. The detailed definition of clinical outcomes (MI, stroke, ICH and GIB) was summarized in S2 Table and was validated in previous study [16]. In brief, MI and GIB were defined as primary discharge diagnostic codes at hospitalization. Stroke and ICH were defined as primary discharge diagnostic codes and claims of brain imaging studies such as computed tomography or magnetic resonance imaging at hospitalization. The index date was defined as the date when the patients had PCI with stent implantation. Patients were censored at the outcome events or at the end of the study period (December 31, 2015), whichever comes first.

Baseline characteristics were presented as mean ± standard deviation and numbers with percentages for continuous variables and categorical variables respectively. The difference in baseline characteristics between groups was compared using student t test or one-way ANOVA for continuous variable and chi-square test for categorical variables. Ischaemic, bleeding and composite clinical outcomes were compared between patients with (1) OAC and APT only, (2) OAC ± SAPT and TT, and (3) OAC ± SAPT and APT only. For the comparison between the groups, we demonstrated a propensity score matching analysis [17]. The propensity score in each comparison was estimated with a logistic regression model based on the covariates: age, sex, DM, dyslipidemia, CHF, peripheral arterial disease, previous myocardial infarction, previous history of PCI, previous history of ICH or stroke, and CHA_2_DS_2_-VASc score. In each comparison, patients were matched with 1:1 fashion using the greedy nearest-neighbor method with a caliper of 0.01 of the propensity score [17]. Post-matching balance of baseline characteristics between the groups was assessed using the absolute standardized difference (ASD), which the value less than 0.1 indicates a negligible difference [18]. We also performed sensitivity analysis using three different multivariable Cox hazard regression model based on the covariates (Model 1: age and sex; Model 2: age, sex, and CHA_2_DS_2_-VASc score; Model 3: age, sex, CHA_2_DS_2_-VASc score, DM, hypertension, dyslipidemia, previous history of CHF, stroke or systemic thromboembolism, MI, PAD, PCI, and ICH). Statistical significance was set at two-tailed p values of <0.05. Statistical analyses were performed using the SAS software version 9.3 (SAS Institute, Cary, NC, USA).

## Results

**Fig 1:**
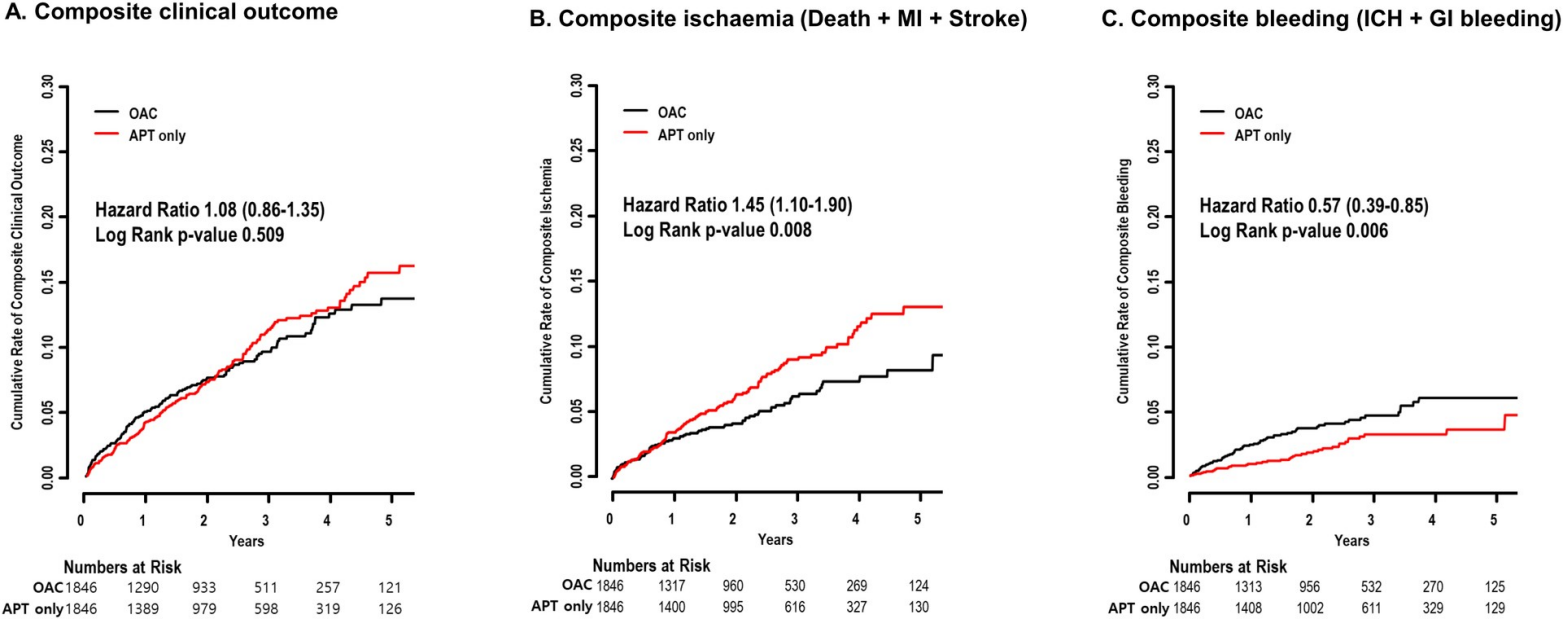
Ischaemic/bleeding risk in patients with AF according to OAC prescription at 1 year after PCI. Cumulative risk of composite clinical outcome (Fig 1A), composite ischaemia (Fig 1B), and composite bleeding (Fig 1C) was compared between patients with OAC and APT only. AF = atrial fibrillation, APT = antiplatelets, GI = gastrointestinal, ICH = intracranial hemorrhage, MI = myocardial infarction, OAC = oral anticoagulants, PCI = percutaneous coronary intervention.

Of total population (N = 13,278), 1,846 (13.9%) received OAC at 1-year after PCI. Patients with OAC were older, had a higher prevalence of male, DM, CHF, previous history of stroke and a higher CHA_2_DS_2_-VASc score compared to those with APT only (Table 1). After 1:1 propensity score matching, the difference in baseline characteristics was well balanced (ASD <0.1) and total 1,846 pairs of patients were included for analysis. The mean follow-up duration was 2.5 years. There was no significant difference regarding composite clinical outcome between patients with OAC and APT only (hazard ratio [HR] 1.08, confidence interval [CI] 0.86–1.35) (Fig 1A, Table 2). Those with APT only had a higher risk of composite ischaemic events (HR 1.45, 95% CI 1.10–1.90) (Fig 1B) with a lower risk of composite bleeding (HR 0.57, 95% CI 0.39–0.85) (Fig 1C). Similar results were found by sensitivity analysis using multivariable Cox regression analysis (S3 Table).

**Table 1:** Baseline characteristics of study population according to OAC prescription at 1-year after PCI.

	Crude population			PS Matched population		
	OAC ± APT	APT only	p-value	OAC ± APT	APT only	ASD
	(N = 1,846)	(N = 11,432)		(N = 1,846)	(N = 1,846)	
**Age, years**	68.9 ± 8.9	68.2 ± 10.2	0.005	68.9 ± 8.9	69.0 ± 9.4	0.012
**Age (≥ 65)**	1,349 (73.1)	7,806 (68.3)	<0.001	1,349 (73.1)	1,355 (73.4)	0.007
**Male**	1,288 (69.8)	7,218 (63.1)	<0.001	1,288 (69.8)	1,292 (70.0)	0.005
**Diabetes mellitus**	723 (39.2)	4,166 (36.4)	0.024	723 (39.2)	745 (40.4)	0.024
**Hypertension**	1,639 (88.8)	10,080 (88.2)	0.448	1,639 (88.8)	1,655 (89.7)	0.028
**Dyslipidemia**	1,501 (81.3)	9,677 (84.7)	<0.001	1,501 (81.3)	1,513 (82.0)	0.017
**Congestive heart failure**	870 (47.1)	4,167 (36.5)	<0.001	870 (47.1)	887 (48.1)	0.018
**Peripheral arterial disease**	382 (20.7)	2,773 (24.3)	<0.001	382 (20.7)	376 (20.4)	0.008
**Previous myocardial infarction**	480 (26.0)	3,086 (27.0)	0.372	480 (26.0)	499 (27.0)	0.023
**Previous PCI**	189 (10.2)	1,613 (14.1)	<0.001	189 (10.2)	164 (8.9)	0.046
**Previous ICH**	11 (0.6)	107 (0.9)	0.149	11 (0.6)	11 (0.6)	<0.001
**Previous stroke**	673 (36.5)	2,230 (19.5)	<0.001	673 (36.5)	649 (35.2)	0.027
**CHA**_**2**_**DS**_**2**_**-VASc score**	4.75 ± 1.81	4.34 ± 1.82	<0.001	4.75 ± 1.81	4.76 ± 1.83	0.004
0	3 (0.2)	63 (0.6)		3 (0.16)	2 (0.11)	
1	41 (2.2)	481 (4.2)		41 (2.22)	40 (2.17)	
≥ 2	1,802 (97.6)	10,888 (95.2)		1,802 (97.6)	1,804 (97.7)	
**Antithrombotic therapy**						
**Triple therapy**	746 (40.4)	-		746 (40.4)	-	
**Warfarin-based**	740 (40.1)	-		740 (40.1)	-	
**NOAC-based**	6 (0.3)	-		6 (0.3)	-	
**OAC + SAPT**	919 (49.8)	-		919 (49.8)	-	
**Warfarin + Aspirin**	390 (21.1)	-		390 (21.1)	-	
**Warfarin + Clopidogrel**	505 (27.4)	-		505 (27.4)	-	
**NOAC + Aspirin**	9 (0.5)	-		9 (0.5)	-	
**NOAC + Clopidogrel**	15 (0.8)	-		15 (0.8)	-	
**OAC monotherapy**	181 (9.8)	-		181 (9.8)	-	
**Warfarin**	164 (8.9)	-		164 (8.9)	-	
**NOAC**	17 (0.9)	-		17 (0.9)	-	
**DAPT**	-	7,978 (69.8)		-	1,248 (67.6)	
**SAPT**	-	3,454 (30.2)		-	598 (32.4)	
**Aspirin**	-	1,643 (14.4)		-	247 (13.4)	
**Clopidogrel**	-	1,811 (15.8)		-	351 (19.0)	

The numbers are presented as mean ± standard deviation or numbers (percentage) otherwise mentioned.

Abbreviation: ASD, absolute standardized difference; APT, antiplatelets; ICH, intracranial hemorrhage; NOAC, non-Vitamin K antagonist oral anticoagulants, OAC, oral anticoagulants; PCI, percutaneous coronary intervention; PS, propensity score.

**Table 2:** Clinical outcome according to OAC prescription at 1-year after PCI.

	OAC ± APT (N = 1,846)			APT only (N = 1,846)			
	Event, N	Person-year	Rate*	Event, N	Person-year	Rate*	HR (95% CI)
**Composite Clinical Outcome**	143	3972.4	3.6	165	4287.4	3.8	1.08 (0.86–1.35)
**Composite Ischaemic Outcome**	84	4063.4	2.1	129	4344.7	3.0	1.45 (1.10–1.90)
**Death**	17	4157.5	0.4	83	4416.2	1.9	4.63 (2.75–7.81)
**Myocardial Infarction**	23	4129.6	0.6	23	4383.6	0.5	0.95 (0.53–1.69)
**Stroke**	66	4091.3	1.6	80	4377.4	1.8	1.14 (0.82–1.58)
**Composite Bleeding Outcome**	64	4059.6	1.6	39	4357.7	0.9	0.57 (0.39–0.85)
**ICH**	14	4132.5	0.3	13	4403.2	0.3	0.88 (0.41–1.87)
**Gastrointestinal Bleeding**	56	4081.5	1.4	31	4370.7	0.7	0.52 (0.34–0.81)

Abbreviation: APT, antiplatelets; CI, confidence interval; HR, hazard ratio; ICH, intracranial hemorrhage; OAC, oral anticoagulants; PCI, percutaneous coronary intervention.

*100-person years.

Regarding antithrombotic regimens, 181 (1.4%), 919 (6.9%), 746 (5.6%), and 11,432 (86.1%) received OAC monotherapy, OAC + SAPT, TT, and APT only, respectively at 1-year after PCI. (S4 Table) Compared to patients with OAC±SAPT, those with TT were younger and had a higher prevalence of previous history of MI, and PCI event, whereas a lower prevalence of previous history of stroke and CHA_2_DS_2_-VASc score. The baseline characteristics were well balanced between groups after 1:1 propensity score matching (ASD <0.1) resulting in 732 pairs of patients for analysis (Table 3). compared to patients with APT only, those with OAC±SAPT were older and had a higher prevalence of DM, hypertension, CHF, stroke and higher CHA_2_DS_2_-VASc, whereas a lower prevalence of previous MI, PAD and PCI event. After propensity score matching, the baseline characteristics was well balanced (ASD <0.1) and total 1,100 pairs of patients were included (Table 3).

**Table 3:** Characteristics of propensity score matched population according to antithrombotic regimen at 1-year after PCI.

	OAC ± SAPT	TT	ASD	OAC ± SAPT	APT only	ASD
	(N = 732)	(N = 732)		(N = 1,100)	(N = 1,100)	
**Age, years**	68.5 ± 8.9	68.2 ± 9.4	0.032	69.5 ± 8.4	69.7 ± 8.6	0.017
**Age (≥ 65)**	518 (70.8)	516 (70.5)	0.006	831 (75.6)	847 (77.0)	0.034
**Male**	499 (68.1)	522 (71.3)	0.068	755 (68.6)	755 (68.6)	<0.001
**Diabetes mellitus**	295 (40.3)	284 (38.8)	0.031	434 (39.5)	429 (39.0)	0.009
**Hypertension**	653 (89.2)	649 (88.7)	0.017	978 (88.9)	1,004 (91.3)	0.079
**Dyslipidemia**	591 (80.7)	593 (81.0)	0.007	900 (81.8)	918 (83.5)	0.043
**Congestive heart failure**	352 (48.1)	352 (48.1)	<0.001	512 (46.6)	527 (47.9)	0.027
**Peripheral arterial disease**	153 (20.9)	159 (21.7)	0.020	221 (20.1)	213 (19.4)	0.018
**Previous myocardial infarction**	198 (27.1)	204 (27.9)	0.018	271 (24.6)	263 (23.9)	0.017
**Previous PCI**	76 (10.4)	80 (10.9)	0.018	107 (9.7)	91 (8.3)	0.051
**Previous ICH**	3 (0.4)	3 (0.4)	<0.001	8 (0.7)	7 (0.6)	0.011
**Previous stroke**	237 (32.4)	246 (33.6)	0.026	423 (38.5)	406 (36.9)	0.032
**CHA**_**2**_**DS**_**2**_**-VASc score**	4.81 ± 1.81	4.7 ± 1.78	0.061	4.79 ± 1.82	4.78 ± 1.81	0.006
0	1 (0.1)	1 (0.1)		2 (0.18)	2 (0.2)	
1	18 (2.5)	18 (2.5)		21 (1.9)	20 (1.8)	
≥ 2	713 (97.4)	713 (97.4)		1,077 (97.9)	1,078 (98.0)	

The numbers are presented as mean ± standard deviation or numbers (percentage) otherwise mentioned.

Abbreviation: ASD, absolute standardized difference; APT, antiplatelets; ICH, intracranial hemorrhage; NOAC, non-Vitamin K antagonist oral anticoagulants, OAC, oral anticoagulants; PCI, percutaneous coronary intervention; SAPT, single antiplatelet therapy; TT, triple therapy.

**Fig 2:**
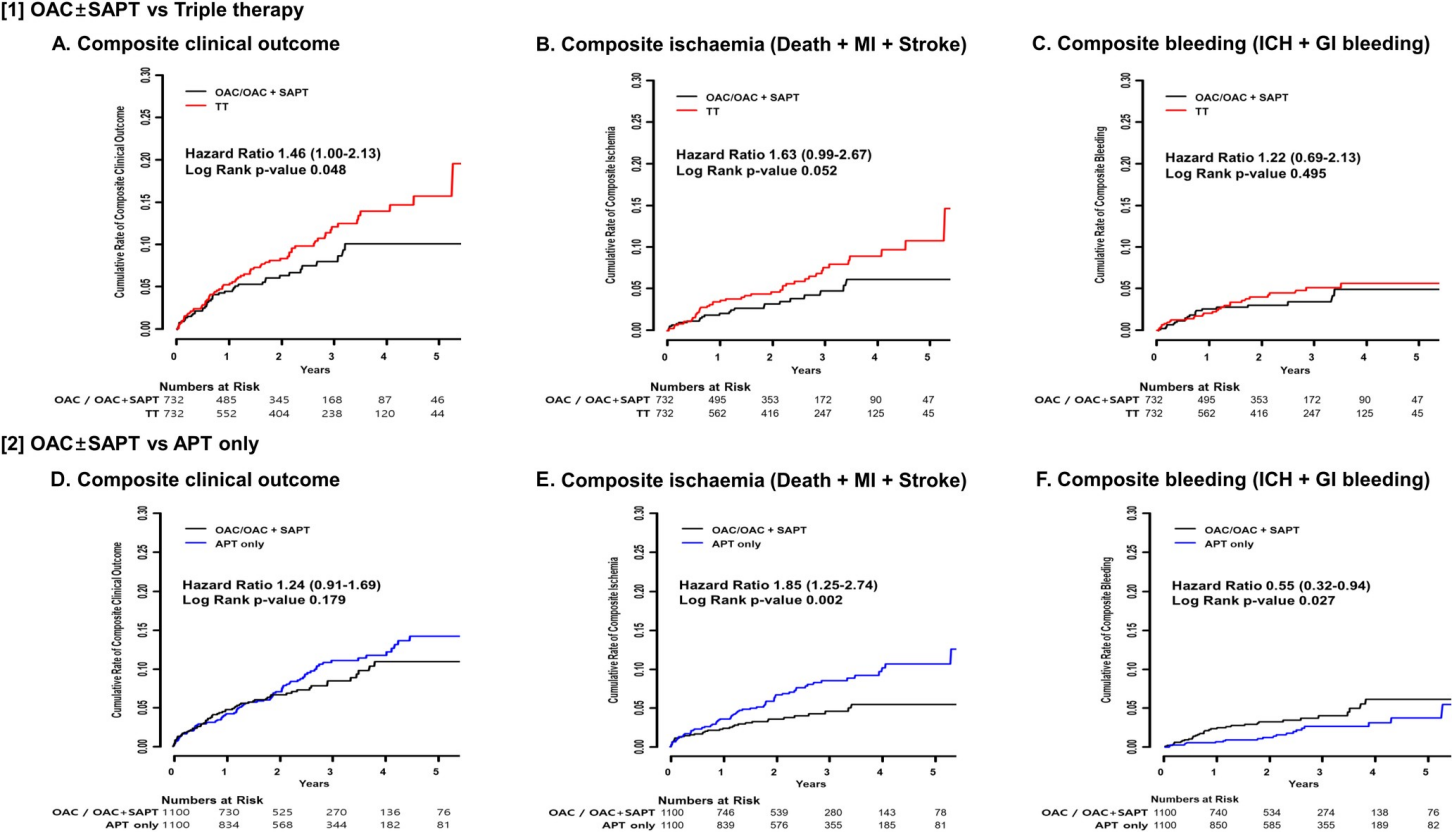
Ischaemic/bleeding risk in patients with AF according to antithrombotic therapy at 1 year after PCI. Cumulative risks for ischaemic, bleeding events, and composite outcome were assessed among patients with TT and APT only compared to those with OAC ± SAPT. AF = atrial fibrillation, APT = antiplatelets, GI = gastrointestinal, ICH = intracranial hemorrhage, MI = myocardial infarction, OAC = oral anticoagulants, PCI = percutaneous coronary intervention, SAPT = single antiplatelets, TT = triple therapy.

Patients with TT showed a significantly higher risk of the composite clinical outcome (HR 1.46, 95% CI 1.00–2.13) (Fig 2A, Table 4) compared to those with OAC±SAPT. This result is attributed by a nonsignificant trend towards a higher risk of composite ischaemic events (HR 1.63, 95% CI 0.99–2.67) (Fig 2B) and composite bleeding (HR 1.22, 95% CI 0.69–2.13) (Fig 2C) in patients with TT compared to those with OAC±SAPT. The results were consistent in the sensitivity analysis using multivariable Cox regression analysis. (S5 Table) Patients with APT only showed a comparable risk of composite clinical outcome compared to those with OAC±SAPT (HR 1.24, 95% CI 0.91–1.69) (Fig 2D, Table 4). This result was driven by a higher risk of composite ischaemia in patients with APT only compare to those with OAC±SAPT (HR 1.85, 95% CI 1.25–2.74) (Fig 2E), which was compensated by a lower risk of composite bleeding (HR 0.55, 95% CI 0.32–0.94) (Fig 2F). Similar trends were found for patients with OAC+SAPT compared to those with APT only (S6 Table). Sensitivity analysis also showed consistent results (S5 Table).

**Table 4:** Clinical outcome according to antithrombotic therapy at 1-year after PCI.

	**OAC** ± **SAPT (N = 732)**			**TT (N = 732)**			
	**Event, N**	**Person-year**	Rate*	**Event, N**	**Person-year**	Rate*	**HR (95% CI)**
**Composite Clinical Outcome**	43	1481.4	2.9	71	1697.5	4.2	1.46 (1.00–2.13)
** Composite Ischaemic Outcome**	24	1508.3	1.6	45	1739.9	2.6	1.63 (0.99–2.67)
** Death**	5	1529.5	0.3	3	1804.6	0.2	0.53 (0.13–2.23)
** Myocardial Infarction**	7	1525.5	0.5	13	1782.6	0.7	1.57 (0.62–3.93)
** Stroke**	21	1512.3	1.4	37	1762.0	2.1	1.55 (0.90–2.67)
** Composite Bleeding Outcome**	21	1500.9	1.4	29	1757.2	1.7	1.22 (0.69–2.13)
** ICH**	6	1521.8	0.4	4	1792.3	0.2	0.60 (0.17–2.13)
** Gastrointestinal Bleeding**	17	1508.6	1.1	25	1769.4	1.4	1.28 (0.69–2.37)
	**OAC** ± **SAPT (N = 1,100)**			**APT only (N = 1,100)**			
	**Event, N**	**Person-year**	Rate*	**Event, N**	**Person-year**	Rate*	**HR (95% CI)**
**Composite Clinical Outcome**	69	2250.9	3.1	94	2521.3	3.7	1.24 (0.91–1.69)
** Composite Ischaemic Outcome**	37	2297.0	1.6	74	2548.7	2.9	1.85 (1.25–2.74)
** Death**	13	2326.0	0.6	43	2601.2	1.7	3.03 (1.63–5.63)
** Myocardial Infarction**	10	2320.2	0.4	19	2571.5	0.7	1.72 (0.80–3.71)
** Stroke**	28	2302.8	1.2	49	2578.4	1.9	1.61 (1.01–2.56)
** Composite Bleeding Outcome**	34	2278.2	1.5	21	2573.8	0.8	0.55 (0.32–0.94)
** ICH**	10	2313.3	0.4	4	2595.8	0.2	0.36 (0.11–1.14)
** Gastrointestinal Bleeding**	30	2287.8	1.3	18	2579.2	0.7	0.53 (0.30–0.95)

Abbreviation: APT, antiplatelets; CI, confidence interval; HR, hazard ratio; ICH, intracranial hemorrhage; OAC, oral anticoagulants; PCI, percutaneous coronary intervention; SAPT, single antiplatelet therapy; TT, triple therapy.

*100-person years.

## Discussion

The current study is the largest nationwide study investigating prescription patterns of antithrombotic therapy in Asian patients with AF at 1-year after PCI and their prognostic impact on long-term ischaemic and bleeding risk. Our principal findings are as follows: (i) most patients (86.1%) did not receive OAC at 1-year after PCI though most of them were clinically indicated for anticoagulation (95.2% with CHA_2_DS_2_-VASc score ≥2); (ii) only a minority of patients (1.4%) received OAC monotherapy at 1-year after PCI, different from guideline recommendations; (3) patients with OAC were associated with a lower risk of composite ischaemic events despite a higher bleeding risk compared to those with APT only; and (iv) patients with APT only were associated with a significantly higher risk of the composite ischaemic outcome and a lower risk of the composite bleeding outcome, when compared to those with OAC±SAPT.

Previous studies had reported under-prescription of OAC among patients with AF beyond 1-year after PCI [17, 18]. In a Danish study reporting antithrombotic therapy among 8,700 patients with AF and stable CAD, more than half of patients (62.7%) had only APT without OAC at 1-year after PCI, whereas only 950 (10.5%) patients received OAC monotherapy [19]. In a recent Asian study, OAC prescription in AF patients at 1-year after PCI was only 2.2%, and most of patients were still maintained on aspirin and clopidogrel (96.1% and 76.3%, respectively) [20]. In line with previous reports, we found that most patients received only APT without OAC despite their high risk of stroke. Considering that current guidelines recommending life-long anticoagulation in patients with AF beyond 1-year after PCI, most of patients in the real world were not in accord with guidelines.

In our results, patients with APT only had a higher prevalence of cardiovascular disease such as MI, PAD, and previous PCI history which would clinically require long-term APT therapy. Previously, we reported similar trends in antithrombotic therapy after PCI among the patients with AF. The prescription rate of TT after PCI was less than 40% in recent 10 years, whereas most patients received DAPT without OAC. Taken together, our results suggest that physicians may opt to maintain APT instead of OAC after PCI in patients with AF. This would often be associated with the clinical ineligibility for OAC treatment such as inadequate international normalized ratio monitoring or concerns regarding a higher risk of bleeding event following combination antithrombotic therapy with both OAC and APT [21]. However, as concomitant history of PAD or CAD significantly increases cardiovascular events in patients with AF [22], optimized combination antithrombotic therapy is essential for these patients.

For patients with AF who had PCI with coronary stent implantation, life-long anticoagulation is recommended in guidelines, after a period of combination antithrombotic therapy, though concomitant SAPT could be considered in those with a high risk of ischaemia, for example, left main coronary artery disease. We found that among 1,846 patients with AF who received OAC at 1-year after PCI, 40.4% of these were still maintained on TT; however, patients on TT showed no clinical benefit compared to those with OAC±SAPT.

Similar results have been reported in non-Asian population. For example, Lamberts et al. investigated the prognostic relevance of antithrombotic regimens among patients with AF and stable CAD [19]. Compared to OAC monotherapy, there was no prognostic benefit in reducing the risk of thromboembolism with combination antithrombotic therapy, but the risk of bleeding was highest in those with TT.

As the numbers of patients with OAC monotherapy was relatively small (N = 181) compared to previous studies, we could not evaluate the prognostic benefit of OAC monotherapy compared to other groups. However, this result could be attributed to a higher percentage of patients who underwent PCI with coronary stent implantation in our study population (100% vs 32.5%), which generally encourages many Korean physicians to maintain prolonged APT without OAC beyond 1-year after PCI. Previously, the Optimizing Antithrombotic Care in Patients With AtriaL fibrillatiON and Coronary stEnt (OAC-ALONE), a prospective randomized controlled open label trial was conducted comparing the efficacy and safety benefit of OAC alone versus OAC plus single APT in AF patients beyond 1-year after PCI with coronary stent implantation [10]. Although no significant differences were observed in primary outcomes (all-cause death, myocardial infarction, stroke, or system embolism) between groups (15.7% vs. 13.6% for OAC monotherapy vs. OAC + SAPT; HR 1.16, 95% CI 0.79–1.72; P for superiority = 0.45; P for non-inferiority = 0.20), the study was underpowered as the trial was prematurely terminated due to delayed enrollment (696 patients in 38 months). Of note that the median interval between subject enrollment and index PCI procedure was 4.4 to 4.6 years, but over 85% of patients in both groups still received concomitant aspirin with OAC at baseline, similar to our results. The addition of SAPT with OAC compared to OAC monotherapy in patients with AF is questionable, as nearly 30% received bare metal stent and those with left main coronary stenting was below 7% of the total trial population.

In the Atrial fibrillation Clopidogrel Trial with Irbesartan for prevention of Vascular Events (ACTIVE W) trial [23]. OAC was superior to clopidogrel plus aspirin in reducing vascular events in patients with AF at high risk of stroke, with no difference in major bleeding between OAC and aspirin-clopidogrel. In patients with CAD, sufficient inhibition of platelet function by DAPT for at least 6 to 12-months after PCI with drug-eluting stent implantation would suffice [24]. However, the benefit of APT would be limited to an initial period of stabilization of the coronary lesion with intensive combination antithrombotic therapy beyond which a sustained hypercoagulable status due to AF [25] persists and requires OAC. In the current study, compared to patients with OAC±SAPT, those with APT only was related to a higher risk of composite ischaemia and stroke suggesting the importance of prolonged OAC in reducing thromboembolic risk among patients with AF after PCI.

An increasing risk of fatal bleeding is the major hurdle in combining OAC prescription on APT therapy [21]. In the current study, the benefit of OAC in reducing the ischaemic risk was compensated by a higher risk of bleeding events, especially for GI bleeding. The higher bleeding risk in patients with OAC groups compared to those with APT only would be accentuated by prolonged prescription of APT in these patients, which is inconsistent with guideline recommendations [1, 2]. Since NOAC has been the primary choice for anticoagulation in AF management [1–3], growing evidence has shown the clinical benefit of NOAC-based combination therapy after PCI in AF patients compared with conventional VKA-based triple therapy. Currently, 4 randomized trials with different types of NOAC have been reported: PIORNEER AF-PCI (rivaroxaban) [26], RE-DUAL PCI (dabigatran) [27], AUGUSTUS (apixaban) [28], and ENTRUST-AF PCI (edoxaban) [29]. These trials have confirmed that NOAC-based dual therapy was superior for bleeding risks and comparable for ischaemic risks as stroke and adverse cardiovascular events than VKA-based triple therapy. In our result, most of the patients with OAC had prescribed warfarin, which may attribute to an even higher risk for bleedings in the OAC group than that would be expected with NOAC. Considering that the proportions for NOAC are also expected in the long-term antithrombotic therapy after PCI [5, 6], future nationwide studies with extended inclusion period would be valuable demonstrating the effectiveness and safety of NOAC-based antithrombotic therapy in AF patients beyond 1-year after PCI.

There are several limitations to our study. First, because of the non-randomized design of the current study, there could be potential confounding factors despite propensity score matching between groups. Second, we categorized the patients according to the antithrombotic regimen at 1-year after PCI. However, the data regarding the temporal change in antithrombotic therapy were not included in our results. Third, as the study population and clinical data were derived from the NHIS claim database, the information regarding drug compliance with laboratory results such as INR could not be obtained. Fourth, detailed information on coronary lesion characteristics including left main coronary disease or multi-vessel disease and procedural results were not available in the database. Fifth, as the number of patients was small, we could not assess the prognostic impact of OAC monotherapy related to long-term ischaemia/bleeding risk in patients with AF after PCI. Last, the data regarding individual risk of bleeding (i.e. HAS-BLED score or previous history of bleeding) were not available in our database.

## Conclusions

OAC prescription was low at 1-year after PCI in patients with AF, and substantial proportion of them had TT. OAC monotherapy with or without SAPT was associated with better clinical outcomes compared to TT or APT treatments, beyond 1-year after PCI among Asians with AF.

## Supporting information

S1 Table Definition of comorbidities. (PDF)

S2 Table Definition of clinical outcomes. (PDF)

S3 Table Sensitivity analysis of ischaemic/bleeding risk in patients with AF according to OAC prescription at 1 year after PCI. (PDF)

S4 Table Baseline characteristics of study population according to antithrombotic regimen at 1-year after PCI. (PDF)

S5 Table Sensitivity analysis of ischaemic/bleeding risk in patients with AF according to antithrombotic regimen at 1 year after PCI. (PDF)

S6 Table Clinical outcome according to antithrombotic therapy at 1-year after PCI (OAC + SAPT vs. APT only). (PDF)

S1 Fig Study flow. Of 226,118 patients who had PCI with coronary stent implantation from 2009 to 2013, a total of 13,278 patients with NVAF and complete prescription records of antithrombotic therapy at 1-year after PCI were included. Patients were categorized based on the prescription regimen of antithrombotic therapy (OAC ± SAPT, TT, and APT only group). Propensity score matching technique (1:1 fashion) was conducted between treatment groups. AF = atrial fibrillation, APT = antiplatelets, DAPT = dual antiplatelets, NVAF = non-valvular AF, OAC = oral anticoagulants, SAPT = single antiplatelets, PCI = percutaneous coronary intervention, PS = propensity score, TT = triple therapy.
(PDF)
