# Supplementary material for: Outcomes in relation to antithrombotic therapy among patients with atrial fibrillation after percutaneous coronary intervention
Source: PLoS One. 2020 Oct 15;15(10):e0240161. doi: 10.1371/journal.pone.0240161 (PMC7561121; doi:10.1371/journal.pone.0240161)
Supplement: S2 Table — (PDF) [file pone.0240161.s002.pdf]

S2 Table. Definition of clinical outcomes

Clinical Outcomes	Primary discharge diagnostic codes /Procedure codes	Definition
**Myocardial infarction**	I21, I22	Primary discharge diagnostic codes
**Ischemic stroke**	I63, I64	Primary discharge diagnostic codes + Claims of brain imaging studies (CT or MRI)
**Gastrointestinal bleeding**	K22.6, K25.0, K25.2, K25.4, K25.6, K26.0, K26.2, K26.4, K26.6, K27.0, K27.2, K27.4, K27.6, K28.0, K28.2, K28.4, K28.6, K29.0, K62.5, K92.0, K92.1, K92.2	Primary discharge diagnostic codes
**Intracranial hemorrhage**	I60, I61, I62	Primary discharge diagnostic codes + Claims of brain imaging studies (CT or MRI)

Abbreviation: CT, computed tomography; MRI, magnetic resonance imaging; PCI, percutaneous coronary intervention.
