# Supplementary material for: Outcomes in relation to antithrombotic therapy among patients with atrial fibrillation after percutaneous coronary intervention
Source: PLoS One. 2020 Oct 15;15(10):e0240161. doi: 10.1371/journal.pone.0240161 (PMC7561121; doi:10.1371/journal.pone.0240161)
Supplement: S3 Table — (PDF) [file pone.0240161.s003.pdf]

S3 Table 3. Sensitivity analysis of ischaemic/bleeding risk in patients with AF according to OAC prescription at 1 year after PCI

	Event, N	Person-year	Rate*	Model 1†	Model 2‡	Model 3§
**Death**						
OAC	17	4157.5	0.4	1	1	1
APT only	426	28422	1.5	3.63 (2.24-5.90)	3.818 (2.35-6.21)	4.08 (2.50-6.64)
**MI**						
OAC	23	4129.6	0.6	1	1	1
APT only	171	28144.8	0.6	1.14 (0.74-1.76)	1.28 (0.82-1.98)	1.18 (0.76-1.84)
**Stroke**						
OAC	66	4091.3	1.6	1	1	1
APT only	425	28233.6	1.5	0.95 (0.73-1.23)	0.99 (0.77-1.29)	1.05 (0.81-1.37)
**ICH**						
OAC	14	4132.5	3.4	1	1	1
APT only	46	28372.5	1.6	0.48 (0.26-0.87)	0.49 (0.27-0.89)	0.53 (0.29-0.98)
**GI Bleeding**						
OAC	56	4081.5	1.4	1	1	1
APT only	181	28150.1	0.6	0.49 (0.36-0.66)	0.50 (0.37-0.67)	0.49 (0.36-0.66)
**Composite Ischaemic Outcome**						
OAC	84	4063.4	2.1	1	1	1

APT only	701	27958.0	2.5	1.23 (0.98-1.54)	1.33 (1.06-1.67)	1.39 (1.10-1.74)
**Composite Bleeding Outcome**						
OAC	64	4059.6	1.6	1	1	1
APT only	213	28101.2	0.8	0.493 (0.37-0.65)	0.50 (0.38-0.67)	0.51 (0.38-0.68)
**Composite Clinical Outcome**						
OAC	143	3972.4	3.6	1	1	1
APT only	884	27651.6	3.2	0.91 (0.76-1.08)	0.97 (0.81-1.16)	1.01 (0.84-1.21)

Abbreviation: AF, atrial fibrillation; APT, antiplatelets; GI, gastrointestinal; ICH, intracranial hemorrhage; MI, myocardial infarction; OAC, oral anticoagulants; PCI, percutaneous coronary intervention.

*100-person years

†Model 1: age and sex

‡Model 2: age, sex, and CHA2DS2-VASc score

§Model 3: age, sex, CHA2DS2-VASc score, diabetes mellitus, hypertension, dyslipidemia, previous history of congestive heart failure, stroke or systemic thromboembolism, MI, PAD, PCI, and ICH
