# Supplementary material for: Outcomes in relation to antithrombotic therapy among patients with atrial fibrillation after percutaneous coronary intervention
Source: PLoS One. 2020 Oct 15;15(10):e0240161. doi: 10.1371/journal.pone.0240161 (PMC7561121; doi:10.1371/journal.pone.0240161)
Supplement: S4 Table — (PDF) [file pone.0240161.s004.pdf]

S4 Table. Baseline characteristics of study population according to antithrombotic regimen at 1-year after PCI

	OAC only (N=181)	OAC + SAPT (N=919)	TT (N=746)	DAPT only (N=7,978)	SAPT only (N=3,454)	p-value
**Age, years**	71.4 ± 8.4	69.1 ± 8.4	68.0 ± 9.6	68.0 ± 10.2	68.6 ± 10.0	<0.001
**Age (≥ 65)**	144 (79.6)	687 (74.8)	518 (69.4)	5,394 (67.6)	2,412 (69.8)	<0.001
**Male**	116 (64.1)	639 (69.5)	533 (71.5)	5,093 (63.8)	2,125 (61.5)	<0.001
**Diabetes mellitus**	72 (39.8)	362 (39.4)	289 (38.7)	2,918 (36.6)	1,248 (36.1)	0.250
**Hypertension**	162 (89.5)	816 (88.8)	661 (88.6)	7,059 (88.5)	3,021 (87.5)	0.542
**Dyslipidemia**	138 (76.2)	762 (82.9)	601 (80.6)	6,803 (85.3)	2,874 (83.2)	<0.001
**Congestive heart failure**	92 (50.8)	420 (45.7)	358 (48.0)	2,900 (36.4)	1,267 (36.7)	<0.001
**Peripheral arterial disease**	37 (20.4)	184 (20.0)	161 (21.6)	1,929 (24.2)	844 (24.4)	0.019
**Previous myocardial infarction**	44 (24.3)	227 (24.7)	209 (28.0)	2,231 (28.0)	855 (24.8)	0.003
**Previous PCI**	25 (13.8)	82 (8.9)	82 (11.0)	1,134 (14.2)	479 (13.9)	<0.001
**Previous ICH**	1 (0.6)	7 (0.8)	3 (0.4)	69 (0.9)	38 (1.1)	0.378
**Previous stroke**	76 (41.99)	347 (37.8)	250 (33.5)	1,503 (18.8)	727 (21.1)	<0.001

**CHA_2DS_2-VASc score**	5.22 ± 1.77	4.71 ± 1.82	4.69 ± 1.79	4.33 ± 1.82	4.37 ± 1.83	<0.001
0	0 (0)	2 (0.2)	1 (0.1)	45 (0.6)	18 (0.5)	
1	3 (1.7)	18 (2.0)	20 (2.7)	316 (4.0)	165 (4.8)	
≥ 2	178 (98.3)	899 (97.8)	725 (97.2)	7,617 (95.4)	3,271 (94.7)	
**NOAC**	17 (9.4)	24 (2.6)	6 (0.8)	-	-	

The numbers are presented as mean ± standard deviation or numbers (percentage) otherwise mentioned.
Abbreviation: APT, antiplatelets; DAPT, dual antiplatelet therapy; ICH, intracranial hemorrhage; NOAC, non-Vitamin K antagonist oral anticoagulants, OAC, oral anticoagulants; PCI, percutaneous coronary intervention; SAPT, single antiplatelet therapy; TT, triple therapy.
