# Supplementary material for: Outcomes in relation to antithrombotic therapy among patients with atrial fibrillation after percutaneous coronary intervention
Source: PLoS One. 2020 Oct 15;15(10):e0240161. doi: 10.1371/journal.pone.0240161 (PMC7561121; doi:10.1371/journal.pone.0240161)
Supplement: S5 Table — (PDF) [file pone.0240161.s005.pdf]

S5 Table. Sensitivity analysis of ischaemic/bleeding risk in patients with AF according to antithrombotic regimen at 1 year after PCI

	Event, N	Person-year	Rate*	Model 1[†]	Model 2[‡]	Model 3[§]
**Death**						
OAC ± SAPT	13	2326.0	0.6	1	1	1
TT	4	1831.5	0.2	0.45 (0.15-1.37)	0.44 (0.14-1.36)	0.45 (0.15-1.38)
APT only	59	20757.8	0.3	0.60 (0.33-1.09)	0.63 (0.35-1.16)	0.67 (0.36-1.24)
**MI**						
OAC ± SAPT	10	2320.2	0.4	1	1	1
TT	13	1809.4	0.7	1.70 (0.74-3.87)	1.65 (0.73-3.77)	1.61 (0.71-3.69)
APT only	133	20484.7	0.6	1.58 (0.83-3.01)	1.73 (0.91-3.30)	1.52 (0.79-2.92)
**Stroke**						
OAC ± SAPT	28	2302.8	1.2	1	1	1
TT	38	1788.5	2.1	1.86 (1.14-3.05)	1.83 (1.12-3.00)	1.90 (1.16-3.11)
APT only	287	20599.4	1.4	1.22 (0.82-1.81)	1.27 (0.85-1.88)	1.39 (0.93-2.07)
**ICH**						
OAC ± SAPT	10	2313.3	0.4	1	1	1
TT	4	1819.2	0.2	0.54 (0.17-1.71)	0.53 (0.17-1.69)	0.57 (0.18-1.84)
APT only	33	20718.5	0.2	0.39 (0.19-0.80)	0.39 (0.19-0.81)	0.45 (0.22-0.93)
**GI Bleeding**						
OAC ± SAPT	30	2287.8	1.3	1	1	1
TT	26	1793.7	1.4	1.15 (0.68-1.94)	1.15 (0.68-1.94)	1.16 (0.69-1.97)

APT only	133	20531.2	0.6	0.52 (0.35-0.78)	0.53 (0.36-0.79)	0.53 (0.36-0.80)
**Composite Ischaemic Outcome**						
OAC ± SAPT	37	2297.0	1.6	1	1	1
TT	47	1766.4	2.7	1.72 (1.12-2.65)	1.70 (1.10-2.61)	1.73 (1.12-2.66)
APT only	701	27958.0	2.5	1.60 (1.15-2.23)	1.72 (1.24-2.40)	1.82 (1.30-2.53)
**Composite Bleeding Outcome**						
OAC ± SAPT	34	2278.2	1.5	1	1	1
TT	30	1781.4	1.7	1.18 (0.72-1.92)	1.17 (0.72-1.92)	1.21 (0.74-1.98)
APT only	159	20492.5	0.8	0.55 (0.38-0.80)	0.56 (0.38-0.81)	0.58 (0.40-0.85)
**Composite Clinical Outcome**						
OAC ± SAPT	69	2250.9	3.1	1	1	1
TT	74	1721.5	4.3	1.46 (1.05-2.03)	1.44 (1.04-2.00)	1.47 (1.06-2.04)
APT only	884	27651.6	3.2	1.08 (0.85-1.38)	1.15 (0.90-1.47)	1.21 (0.94-1.55)

Abbreviation: AF, atrial fibrillation; DAPT, dual antiplatelets; GI, gastrointestinal; ICH, intracranial hemorrhage; MI, myocardial infarction; OAC, oral anticoagulants; PCI, percutaneous coronary intervention; SAPT, single antiplatelets, TT, triple therapy.

*100-person years

†Model 1: age and sex

‡Model 2: age, sex, and CHA2DS2-VASc score

§Model 3: age, sex, CHA2DS2-VASc score, diabetes mellitus, hypertension, dyslipidemia, previous history of congestive heart failure, stroke or systemic thromboembolism, MI, PAD, PCI, and ICH
