# Supplementary material for: Outcomes in relation to antithrombotic therapy among patients with atrial fibrillation after percutaneous coronary intervention
Source: PLoS One. 2020 Oct 15;15(10):e0240161. doi: 10.1371/journal.pone.0240161 (PMC7561121; doi:10.1371/journal.pone.0240161)
Supplement: S6 Table — (PDF) [file pone.0240161.s006.pdf]

S6 Table. Clinical outcome according to antithrombotic therapy at 1-year after PCI (OAC + SAPT vs. APT only)

	OAC + SAPT (N=919)			APT only (N=919)			
	**Event, N**	**Person-year**	**Rate***	**Event, N**	**Person-year**	**Rate***	**HR (95% CI)**
**Composite Clinical Outcome**	61	2022.5	3.0	74	2105.0	3.5	0.86 (0.61-1.20)
**Composite Ischaemic Outcome**	31	2064.1	1.5	59	2121.7	2.8	0.54 (0.35-0.83)
**Death**	10	2087.2	0.5	36	2160.4	1.7	0.29 (0.14-0.58)
**Myocardial Infarction**	8	2082.6	0.4	17	2137.7	0.8	0.48 (0.21-1.12)
**Stroke**	25	2068.8	1.2	39	2144.4	1.8	0.65 (0.39-1.07)
**Composite Bleeding Outcome**	32	2043.8	1.6	16	2143.8	0.7	2.12 (1.16-3.86)
**ICH**	10	2074.5	0.5	5	2158.5	0.2	2.10 (0.72-6.14)
**Gastrointestinal Bleeding**	27	2053.5	1.3	13	2145.8	0.6	2.20 (1.13-4.26)

Abbreviation: APT, antiplatelets; CI, confidence interval; HR, hazard ratio; ICH, intracranial hemorrhage; OAC, oral anticoagulants; PCI, percutaneous coronary intervention; SAPT, single antiplatelets.
*100-person years
