# Supplementary figures and images for: Outcomes in relation to antithrombotic therapy among patients with atrial fibrillation after percutaneous coronary intervention
Source: PLoS One. 2020 Oct 15;15(10):e0240161. doi: 10.1371/journal.pone.0240161 (PMC7561121; doi:10.1371/journal.pone.0240161)

S1 Fig. Study flow

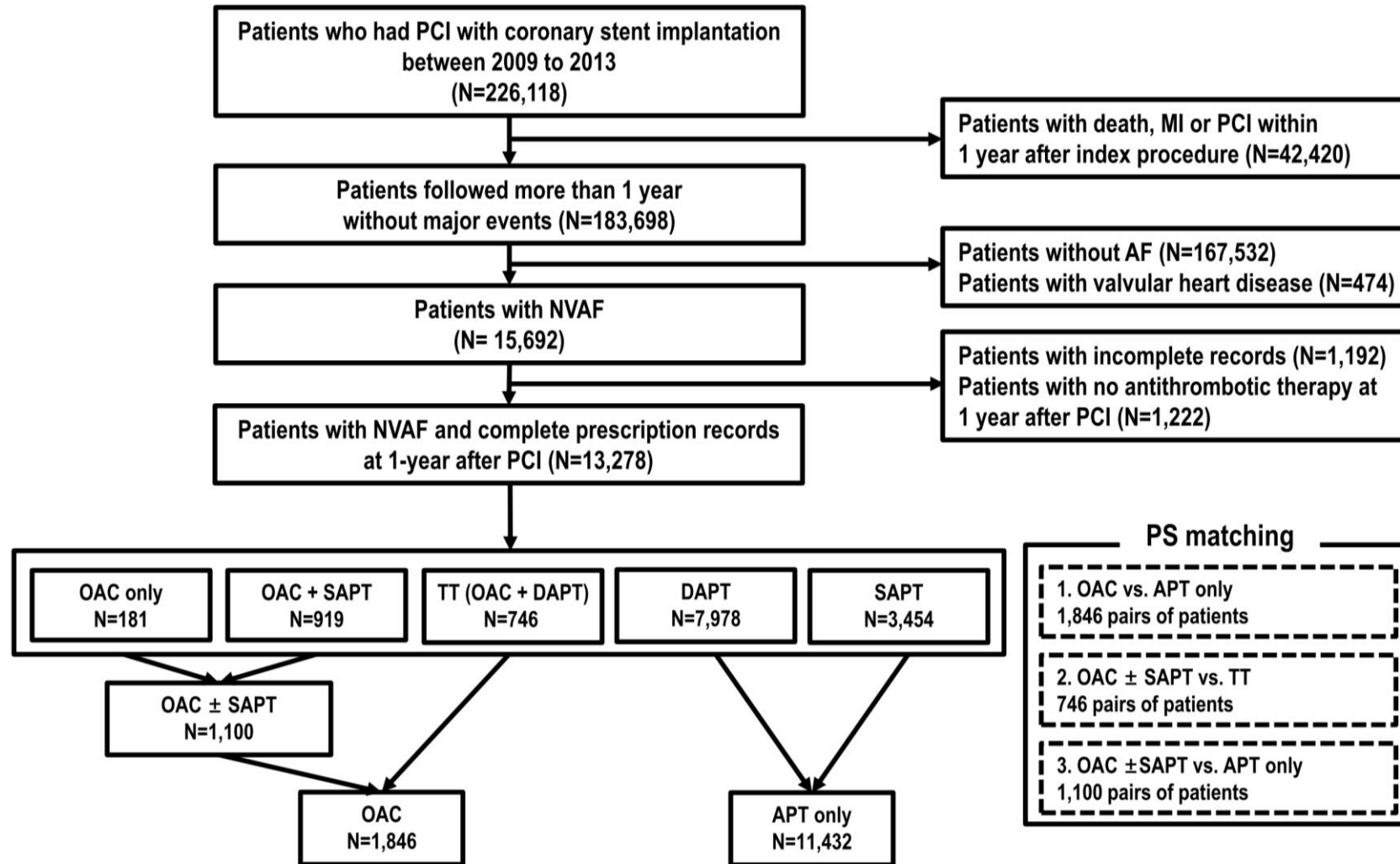

Supplement: S1 Fig — Of 226,118 patients who had PCI with coronary stent implantation from 2009 to 2013, a total of 13,278 patients with NVAF and complete prescription records of antithrombotic therapy at 1-year after PCI were included. Patients were categorized based on the prescription regimen of antithrombotic therapy (OAC ± SAPT, TT, and APT only group). Propensity score matching technique (1:1 fashion) was conducted between treatment groups. AF = atrial fibrillation, APT = antiplatelets, DAPT = dual antiplatelets, NVAF = non-valvular AF, OAC = oral anticoagulants, SAPT = single antiplatelets, PCI = percutaneous coronary intervention, PS = propensity score, TT = triple therapy. (PDF) [file pone.0240161.s007.pdf]
